# Sirenomelia in a Nigerian triplet: a case report

**DOI:** 10.1186/1752-1947-5-426

**Published:** 2011-09-02

**Authors:** Rosemary O Ugwu, Augusta U Eneh, Woroma Wonodi

**Affiliations:** 1 Department of Paediatrics and Child Health, University of Port Harcourt Teaching Hospital, Port Harcourt, Nigeria

## Abstract

**Introduction:**

Sirenomelia, also known as mermaid syndrome, is a very rare fatal congenital abnormality in which the legs are fused together, giving them the appearance of a mermaid's tail. It is commonly associated with abnormal kidney development, genital and rectal abnormalities. A handful of cases have been reported in other parts of the world, however, no cases have previously been reported in a Nigerian neonate. To the best of our knowledge, we believe that this is the first case reported from West Africa and in a triplet.

**Case presentation:**

A 16-hour-old baby boy, the second of a set of Nigerian triplets, presented to our facility with fusion of the entire lower limbs, imperforate anus, indiscernible genital structures, single umbilical artery and a neural tube defect. His parents were from the Hausa ethnic group and not related.

**Conclusion:**

Sirenomelia has not been previously described in a set of triplets, and it is hoped that this report from West Africa will give information about the non-racial predilection of this condition.

## Introduction

Sirenomelia is a very rare congenital abnormality in which the legs are fused together, giving them the appearance of a mermaid's tail. This condition is found in approximately 1 in 100,000 live births [1] and is usually fatal [2]. It is commonly associated with renal agenesis, absent or malformed external and internal genitalia, a single umbilical artery, imperforate anus, and a blind ending large intestine [3]. Other abnormalities reported in association include double inferior vena cava [4] and angiomatous lumbosacral myelocystocoele [5].

More than half the cases of sirenomelia result in stillbirth and those born alive usually die within a day or two of birth because of complications associated with abnormal kidney and bladder development and function. Only a handful of patients with sirenomelia have been reported to have survived beyond the neonatal period [6-8]. Few cases have been reported in one baby of a set of twins [3,9]. To the best of our knowledge, there has been no previous report of sirenomelia from Africa or in one baby of a set of triplets. We report the case of a Nigerian triplet of Hausa ethnicity with sirenomelia. The Hausa ethnic group has the lowest twinning rate in Nigeria [10].

## Case presentation

The patient, a 16-hour-old Nigerian baby boy was brought to our children's out-patient clinic on account of fusion of the two legs from birth. The baby was delivered at term by spontaneous vaginal delivery after an unsupervised pregnancy and was the second of a set of triplets. The first triplet, a boy, was alive and well, but the third triplet (also a boy) died soon after birth. The mother was 20 years old and the father 26 years old. Both parents were from Hausa ethnic group, and had no formal education and were unrelated. The mother had no medical illness and did not take fertility drugs. This was her first pregnancy. There was no family history of congenital abnormality.

**Figure 1:**
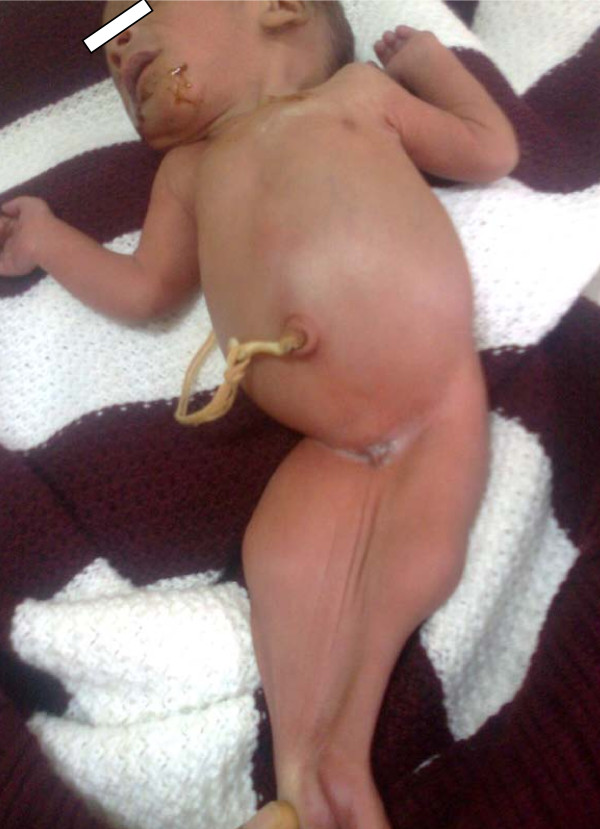
**Anterior view of our patient showing the fused lower limbs (with the thigh and leg bones appearing separate), absent external genitalia and severe talipes equinovarus deformity of the feet**.

**Figure 2:**
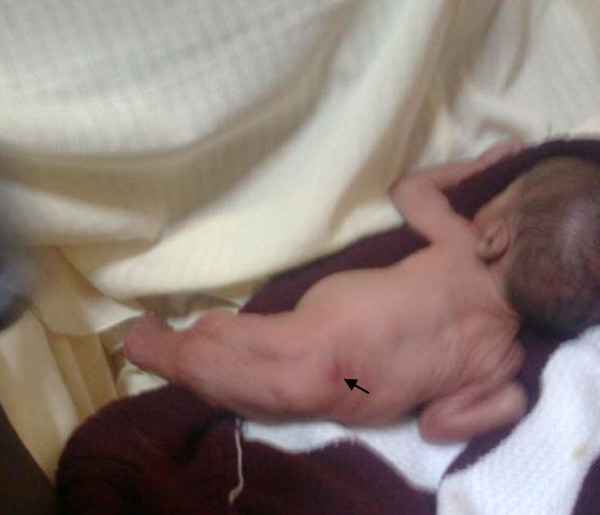
**Posterior view of our patient showing the neural tube defect (arrow) and absent anal opening**.

The baby had a birth weight of 1 kg. There was fusion of the entire lower limbs from the hip to the ankle with bones present in the thighs (femur) and the legs (tibia and fibula). There was no anal opening and no discernable external genital organs (Figures 1 and 2). There was a spinal defect at the level of L2-L3. The umbilical stump revealed only one artery and one vein. The heart sounds were normal. The parents refused any investigation or intervention when the prognosis was explained to them and took the baby away.

## Discussion

The cause of sirenomelia remains unclear, however, maternal diabetes mellitus [3,11,12], genetic predisposition, environmental factors and vascular steal phenomenon with the single vitelline umbilical artery diverting blood supply and nutrients from the lower body and limbs [13] have been proposed as possible causative factors. The pattern of birth defects seen in sirenomelia is associated with abnormal umbilical cord blood vessels. Most babies with sirenomelia have only one umbilical artery and one vein, as was seen in our patient.

The spectrum of malformation of the lower limbs seen in babies with sirenomelia ranges from fusion of the legs into one lower limb with only two bones present in the entire limb (a femur and a tibia) and absence of foot structures to fusion of the skin of the lower limbs along the inner leg with fully formed and separate lower limb bones and fully formed feet which are fused at the ankles. This latter form was the case in our patient.

Confusion still exists on whether sirenomelia is a severe form of caudal regression syndrome and VACTERL ('vertebral defects, anorectal atresia, cardiac abnormalities, tracheo-oesophageal fistula, renal and limb abnormalities') association due to overlapping features. Maternal diabetes has been associated with both caudal regression syndrome and sirenomelia [3,11,12]. The imperforate anus, a neural tube defect at the level of L2-L3, limb abnormalities and presumably, a renal abnormality (because of the single umbilical artery) seen in our patient are all components of VACTERL association.

The diagnosis is obvious at birth on examination of a baby, but pre-natal diagnosis can also be made as early as the first trimester by an ultrasound [9,14]. This was not possible in our case as the mother's pregnancy was unsupervised. After delivery, an infantogram can show the exact bony abnormalities while abdominal ultrasound can demonstrate abnormalities of the internal organs. These were not performed as the parents strongly refused any investigation or interventions.

## Conclusions

Sirenomelia is a very rare fatal congenital abnormality. To the best of our knowledge, this is the first case reported from West Africa and in a set of triplets. It is our hope that this report will add to existing knowledge and data about the condition. The parent's refusal for investigations limited our description of the full spectrum of sirenomelia in our patient.

## Consent

Written informed consent was obtained from the patient's next-of-kin for publication of this case report and any accompanying images. A copy of the written consent is available for review by the Editor-in-Chief of this journal.

## Competing interests

The authors declare that they have no competing interests.

## Authors' contributions

ROU contributed substantially to the conception, design and acquisition of data and was also a major contributor to the drafting and writing of the manuscript. AUE was involved in drafting the manuscript as well as revising it critically for important intellectual content. WW was involved in the summary of the case as well as obtaining the accompanying images. All authors read and approved the final manuscript.
